# Demyelinating Diseases: From Molecular Mechanisms to Therapeutic Strategies—3rd Edition

**DOI:** 10.3390/ijms27135830

**Published:** 2026-06-28

**Authors:** Antonietta Bernardo

**Affiliations:** National Center for Research and Preclinical and Clinical Evaluation of Drugs, Istituto Superiore di Sanità, 00161 Rome, Italy; antonietta.bernardo@iss.it

Demyelinating diseases damage myelin, the protective sheath surrounding nerve fibres in both the central and peripheral nervous systems which assists in the transmission of nerve signals and in the conservation of energy during the propagation of action potentials. These diseases are highly debilitating and affect millions worldwide. They are classified into types based on their features and causes, including immune-mediated, infectious, metabolic, and hypoxic–ischemic forms. Myelin in the CNS, produced by oligodendrocytes, differs from that in the PNS, produced by Schwann cells [1]. Some diseases primarily target peripheral nerves, while others affect the CNS. Currently, few treatments can fully restore myelin function and nerve conduction [2]. This Special Issue, titled “Demyelinating Diseases: From Molecular Mechanisms to Therapeutic Strategies 3.0” [3], continues a series focused on demyelinating diseases, published in the *International Journal of Molecular Sciences* [4,5,6]. The main aims are to review research on the pathogenic mechanisms of demyelinating diseases and to unify efforts to identify and preclinically validate therapeutic targets. These targets may be used in preventive or curative strategies to improve the quality of life for patients affected by these serious conditions. Through original papers or reviews, the authors of this collection aim to investigate the specific mechanisms underlying demyelination and to discover new pharmacological targets.

This Special Issue features original research by Bardy-Lagarde and colleagues [7] on how oestradiol affects myelin repair. The authors highlight that hormonal status is a vital yet often neglected aspect of therapy for this process. Oestradiol and testosterone have the same effect on remyelination in the mouse CNS but act through different mechanisms, as observed in two models: lysolecithin (LPC) and ovariectomy (removal of the ovaries). In LPC-induced demyelinating lesions, experiments show that oestradiol is a key regulator of repair, as testosterone is in men. In ovariectomized mice, oestradiol treatment restores the astrocyte population and enhances remyelination, presumably by improving astrocytes’ ability to maintain glutamate homeostasis, which is dysregulated under these conditions and thus induces damage to oligodendrocytes, which are particularly vulnerable. Oestradiol-mediated repair occurs without CXCR4 (C-X-C motif chemokine receptor 4, a G protein-coupled receptor that binds CXCL12 (SDF-1)), whereas testosterone-dependent repair involves androgen receptor signalling and CXCR4 induction in astrocytes. Throughout the study, it is confirmed that oestradiol is a crucial component of female-specific CNS remyelination and contributes to the repair process by increasing astrocyte numbers and restoring their functions. The authors therefore suggest that oestrogen could have therapeutic relevance, pending clinical validation, and that only in the future, it could be used as replacement therapy and could be beneficial for women with Multiple Sclerosis (MS) who have low oestrogen levels or are menopausal.

In another innovative paper, Ahmad and colleagues [8] examine how platelets contribute to virus-induced inflammatory demyelinating disease and myocarditis. They reveal their unexpected role in maintaining viral persistence in a mouse model of two organ-specific immune-mediated diseases, Theiler’s murine encephalomyelitis virus (TMEV), which serves as a flexible model for studying both MS and viral myocarditis. TMEV-induced demyelinating disease (TMEV-IDD) in the CNS is a chronic inflammatory condition characterised by persistent viral infection, making it a valuable model for studying human MS, a disease in which the immune system is known to damage myelin.

The researchers found that, beyond their role in blood clotting, platelets can hinder viral clearance. In the models studied, platelet depletion enhanced the immune response against the virus. During infection, changes in platelet gene expression (e.g., MHC class I and interferon-related genes) were observed, and platelets adhered to the vascular endothelium near inflammatory lesions in the CNS and the heart. Using anti-GPIbα antibodies to promote platelet depletion, a significant reduction in viral persistence in the CNS and in the severity of TMEV-IDD and myocarditis was observed, as well as increased interferon-γ production and a higher anti-TMEV IgG2a/IgG1 ratio. The findings suggest that, if platelets help viruses evade the immune response in the CNS by suppressing Th1 responses, then anti-platelet therapies, such as blood thinners or targeted antibodies, could be explored to target viral triggers and prevent disease progression, and may serve as a new therapeutic strategy for virus-induced CNS inflammatory diseases, such as MS and viral myocarditis.

A compelling review explores how short-chain fatty acids (SCFAs) such as butyrate, propionate, and acetate, produced by the gut microbiota during dietary fibre fermentation, affect immune responses, enhance the blood–brain barrier (BBB), and reduce neuroinflammation in MS. Since MS patients often have lower levels of gut microbiota and SCFAs, increasing their production or supplementing them could hold therapeutic potential. It is well established that MS affects immune regulation in three key stages: first, an immune imbalance in which pro-inflammatory cells (Th1 and Th17) outnumber regulatory cells; second, barrier failure allowing autoreactive T cells to cross the BBB; third, neurodegeneration featuring localised inflammation, demyelination, and ongoing axonal damage in the central nervous system. Barcutean and colleagues [9] highlighted that SCFAs act as chemical messengers that enhance the expression of regulatory T cells, which help suppress the overactive Th1/Th17 response. They influence BBB permeability, thereby reducing the entry of autoreactive T cells into the CNS and making it more difficult for inflammatory cells to penetrate the brain. Notably, butyrate and propionate reduce inflammation, influence microglial maturation and function, and decrease their activation in a neurotoxic state, demonstrating potent anti-inflammatory effects in both the gut and the CNS, emphasising that targeting the gut–brain axis via SCFAs is a promising therapy.

On the other hand, Prapas and Anagnostouli [10] examine how MS development is influenced by interactions among antigen presentation, HLA genetics, and macrophage-driven immune responses, collectively contributing to inflammation and neurodegeneration. MS is recognised as a T cell-mediated autoimmune disease in which antigen presentation is crucial. Variations in MHC class II genes affect antigen processing and presentation, thereby influencing susceptibility and disease progression. These immunogenetic factors lead to distinct pathogenic pathways in adult-onset (AOMS) and paediatric-onset (POMS) MS. Antigen presentation via MHC class II molecules such as HLA-DRB1*15:01 plays a key role, as high-risk alleles increase myelin protein binding and promote autoreactive T-cell activation. Meanwhile, macrophages and M1 polarisation are essential in lesion formation and disease progression. In parallel, these immunogenetic factors also modulate innate immunity by influencing macrophage behaviour. The pro-inflammatory M1 phenotype, characterised by aggressive activity in both the periphery and the CNS, is strongly associated with lesion formation and neurodegeneration. The authors suggest that the HLA genotype may therefore affect not only T-cell responses but also macrophage balance, thereby increasing susceptibility to lesion development and reinforcing the role of genetic determinants in disease pathogenesis.

The review further compares AOMS and POMS, highlighting a greater genetic contribution in paediatric cases and underscoring how age-related differences in disease progression may inform more tailored therapeutic strategies. The authors emphasise that MS treatment efficacy is heavily influenced by genetics; for example, HLA-A*03:01 is associated with better responses to Glatiramer Acetate (GA) than to Interferon-beta, with relapse risk reductions of 33–63%. Innovative approaches show promise for drugs that target specific cell types through macrophage reprogramming; converting M1 pro-inflammatory macrophages into M2 regulatory ones may minimise CNS damage. Additionally, synthetic materials with higher HLA-binding affinity than GA are being developed to more effectively induce T-cell inactivation or trigger anti-inflammatory responses. Future personalised treatments could be based on an individual’s HLA genotype, identify biomarkers, improve disease outcome assessment, and potentially offer new therapeutic targets for managing progression and blocking immune attacks on nerves.

Sempik and colleagues’ [11] review highlights that Primary Progressive Multiple Sclerosis (PPMS), a rare MS subtype, is associated with a poor prognosis and diagnostic challenges, underscoring the need to elucidate its molecular mechanisms to enable the development of next-generation therapies. PPMS remains a challenge for neuroimmunology and is characterised by a steady build-up of disability without early relapses and remissions, offering a clear view of pure disease progression. As the authors explain, the disease course is driven by a complex trio: widespread axonal loss, microglial-mediated chronic inflammation occurring behind an intact blood–brain barrier, and a marked failure of natural remyelination. Studying PPMS compels researchers to recognise the limitations of conventional peripheral immunosuppressive treatments and to focus instead on the central nervous system microenvironment. This review effectively links laboratory research to clinical care. It emphasises the shift from subjective clinical observation to objective biomarker measurement for diagnosis and monitoring. The validation of fluid biomarkers such as neurofilament light chain, combined with advanced imaging measures of brain and spinal cord atrophy, provides accurate tools for assessing ongoing damage in real time. Ultimately, these molecular and radiological insights will shape future treatment approaches. Although B-cell depletion with ocrelizumab is a landmark therapy as the first one approved for PPMS, it offers only a partial solution. As highlighted in this review, the future of managing demyelinating diseases depends on neuroprotection and active tissue repair. The ongoing clinical trials discussed by the authors are at the forefront of this transformation. The researchers emphasise that PPMS is not merely a minor, isolated form of MS but rather the key to understanding and overcoming all MS progression. By uncovering the molecular mechanisms underlying remyelination failure in PPMS, researchers worldwide can develop targeted, personalised therapies to halt neurodegeneration.

## Conclusions

Demyelinating diseases continue to pose complex biological and clinical challenges while inspiring innovative, multidisciplinary research. This Special Issue offers a comprehensive overview of recent advances in understanding of the mechanisms, therapeutic options, and clinical significance of these diseases. Future research should focus on clarifying causal mechanisms, identifying new metabolites, and translating findings into clinical practice.

## References

[1] Kister A., Kister I. (2023). Overview of Myelin, Major Myelin Lipids, and Myelin-Associated Proteins. Front. Chem..

[2] Medina R., Derias A.-M., Lakdawala M., Speakman S., Lucke-Wold B. (2024). Overview of Emerging Therapies for Demyelinating Diseases. World J. Clin. Cases.

[3] Bernardo A. Special Issue “Demyelinating Diseases: From Molecular Mechanisms to Therapeutic Strategies—3rd Edition”. The Special Issue Belongs to the Section “Molecular Neurobiology”. Int. J. Mol. Sci.

[4] Bernardo A., Visentin S. Special Issue “Demyelinating Diseases: From Molecular Mechanisms to Therapeutic Strategies”. The Special Issue Belongs to the “Molecular Biology” Section. Int. J. Mol. Sci..

[5] Bernardo A., Visentin S. (2023). Demyelinating Diseases: From Molecular Mechanisms to Therapeutic Strategies. Int. J. Mol. Sci..

[6] Bernardo A. Special Issue “Demyelinating Diseases: From Molecular Mechanisms to Therapeutic Strategies 2”. The Special Issue Belongs to the Section “Molecular Neurobiology”. Int. J. Mol. Sci.

[7] Bardy-Lagarde M., Asbelaoui N., Schumacher M., Ghoumari A.M. (2025). Estradiol Promotes Myelin Repair in the Spinal Cord of Female Mice in a CXCR4 Chemokine Receptor-Independent Manner. Int. J. Mol. Sci..

[8] Ahmad I., Omura S., Sato F., Park A.-M., Khadka S., Gavins F.N.E., Tanaka H., Kimura M.Y., Tsunoda I. (2024). Exploring the Role of Platelets in Virus-Induced Inflammatory Demyelinating Disease and Myocarditis. Int. J. Mol. Sci..

[9] Barcutean L., Maier S., Burai-Patrascu M., Farczadi L., Balasa R. (2024). The Immunomodulatory Potential of Short-Chain Fatty Acids in Multiple Sclerosis. Int. J. Mol. Sci..

[10] Prapas P., Anagnostouli M. (2024). Macrophages and HLA-Class II Alleles in Multiple Sclerosis: Insights into Therapeutic Dynamics. Int. J. Mol. Sci..

[11] Sempik I., Dziadkowiak E., Moreira H., Zimny A., Pokryszko-Dragan A. (2024). Primary Progressive Multiple Sclerosis—A Key to Understanding and Managing Disease Progression. Int. J. Mol. Sci..

